# A meta-analysis of perventricular device closure of perimembranous ventricular septal defect

**DOI:** 10.1186/s13019-019-0936-5

**Published:** 2019-06-27

**Authors:** Zhi-Nuan Hong, Qiang Chen, Li-Qin Huang, Hua Cao

**Affiliations:** 1 Department of Cardiac Surgery, Fujian Provincial Maternity and Children’s Hospital, Affiliated Hospital of Fujian Medical University, Fuzhou, 350001 People’s Republic of China; 2 Department of Cardiovascular Surgery, Union Hospital, Fujian Medical University, Fuzhou, 350001 People’s Republic of China; 3 Department of Public Health, Fujian Medical University, Fuzhou, 350001 People’s Republic of China

**Keywords:** Perventricular device closure, Perimembranous ventricular septal defect, Meta-analysis

## Abstract

**Background:**

To investigate the safety and efficacy of perventricular device closure of perimembranous VSD (pmVSD).

**Methods:**

PubMed and Scopus were searched for studies in English focusing on perventricular device closure of pmVSD published up to the end of March 2019. We used a random-effects model to obtain pooled estimates of the success and complication rates.

**Results:**

A total of 15 publications comprising 1368 patients with pmVSD were included. The median follow-up duration was 2 months to 5 years, with a mean patient age ranging from 2 months to 56 years. The pooled success rate was 0.95 (I^2^ = 86.2%, *P* = 0.000). The pooled rate of postoperative residual shunting was 0.02 (95% CI: 0.01–0.03, I^2^ = 87.3%, *P* < 0.001). The pooled rate of residual shunting in the follow-up period was 0.001 (95% CI:-0.001–0.002, I^2^ = 30.5%, *P* = 0.126). The pooled estimated rate of severe complications was 0.074 (95% CI: 0.046–0.102, I^2^ = 30.5%, *P* = 0.126). The pooled incidence of complete atrioventricular block (cAVB) was 0.002 (95% CI: 0.000–0.005, I^2^ = 0.0%, *P* = 0.577).

**Conclusions:**

Perventricular device closure may be an alternative to conventional surgical repair in selected patients with pmVSD. The success rate was stable regarding the publication year and sample size and suggested both the short learning curve of this technology and its potential for wide application. The incidence of severe arrhythmia, especially cAVB, was low. These good results may be limited by the number of enrolled patients, and a more detailed and larger sample is required for further analysis.

## Introduction

Ventricular septal defect (VSD) is one of the most common congenital hearts defects (CHDs), accounting for 20% of all forms of congenital cardiac malformations, and 80% of VSD cases are perimembranous VSD [1, 2]. Conventional surgical repair of VSD under cardiopulmonary bypass (CPB) is the gold standard treatment [3]. However, this approach cannot avoid the potential for CPB-related complications or complete atrioventricular block (cAVB), the surgical incision scar or prolonged recovery [4–7]. With the improvement and development of various devices, transcatheter device closure of pmVSD has also gradually gained popularity in most medical centers with a promising closure success rate [8–10]. Based on the above two methods, perventricular device closure of pmVSD guided by transesophageal/transthoracic echocardiography (TEE/TTE) was developed and has been widely applied in China, with promising results. This study aimed to obtain pooled estimates of the success and morbidity rates after perventricular device closure of pmVSD based on a meta-analysis of the current literature. These clinical data could serve as important evidence for the acceptance of perventricular device closure of pmVSD as an alternative to conventional surgical repair of VSD. This analysis could also guide further research on and development of occluders to achieve better outcomes with fewer complications.

## Methods

### Literature search strategy

A search of the English literature from the start date of each database up to March 2019 was conducted by 2 independent researchers using PubMed (MEDLINE), EMBASE, and the Cochrane Central Register of Controlled Trials with the following search terms: ventricular septal defect, perimembranous, mini-invasive, transthoracic, intraoperative, perventricular, and device closure. From this search list, studies investigating the results of perventricular device closure of pmVSD were identified. Reference lists of the included articles were further examined to identify other relevant studies. Excluded studies and the reasons for their exclusion were listed and examined by a third researcher.

### Study selection and quality assessment

**Fig. 1:**
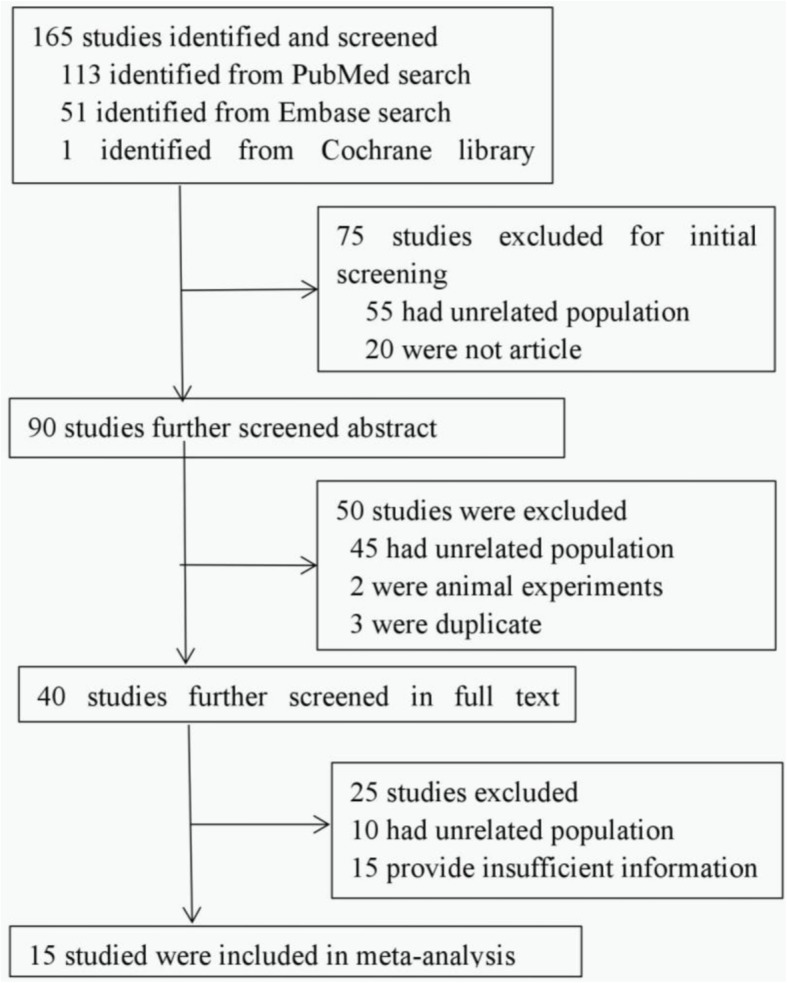
Flow chart of literature selection

The inclusion criteria included studies (randomized and nonrandomized studies) reporting perventricular device closure of congenital pmVSD in humans. The exclusion criteria included case series already included in multi-center studies, case reports with sample sizes less than 10, and reports of acquired pmVSD following myocardial infarction. Our search identified 165 articles, of which 150 were excluded (Fig. 1). A total of 15 articles [11–25] were included and further analyzed. Eight studies were case series, and the other 7 studies were case-control studies. Perventricular device closure was compared with surgical repair in 5 studies, the effectiveness between symmetrical and asymmetrical occluders was compared in 1 study, and guidance with TTE and TEE in regard to feasibility was compared in 1 study.

This meta-analysis included 8 case series and 7 case-control studies. We used the Newcastle-Ottawa Scale (NOS) to assess the quality of the case-control studies. The NOS assesses the quality of studies based on the selection of the cases and controls (0–4 stars), comparability of the cases and controls (0–2 stars) and the ascertainment of exposure (0- 4stars). NOS scores > 6 stars are considered to indicate high quality [26].

We chose an 18-item, validated quality appraisal tool to evaluate the methodological quality of the case series. The quality assessments for each item were binary determinations of various aspects of the study, including the study objective, study population, intervention and cointervention, outcome measures, statistical analysis, results and conclusions, competing interests, and sources of support. High quality scores were ≥ 14 [27]. Disagreements in the quality assessment were resolved through discussion.

### Data extraction

Relevant data were extracted by two authors (Zhi-Nuan Hong and Li-Qin Huang) and entered into an electronic database. The data included publication details, including the publication year and first author, the device type, VSD size, sample size, age, success rate, complications rate, and median follow-up period. Successful device closure was defined as a residual shunt < 2 mm detected by TTE or TEE. Residual shunts, arrhythmias, and valvular lesions were considered permanent if they were reported and remained present at the time of the latest follow-up visit, regardless of severity. Residual shunts included all color jets observed across the VSD after deployment of the device. Complete atrioventricular block was further divided into transient or permanent. Valvular lesions included new-onset, device-related lesions with the exclusion of transient, early lesions that disappeared in the postdeployment period. Data regarding other significant complications, such as device embolization, hemolysis and thromboembolism, were also extracted.

### Statistical analysis

Baseline characteristic data are presented as the median. Zero-event rates were approximated with [1/(4*sample size)] to allow calculation of the pooled occurrence rates. If a particular event was not reported in a study, then this study was excluded from the pooled analysis of these events.

We used a funnel plot of the sample size plotted against the operational success rate to evaluate the possibility of publication bias. The random-effects model was used to obtain the pooled estimates of the success rate and different types of complication rates. This study assumed that the total of 15 studies represented a random sample from the larger population of such studies. Each study had its own underlying effect size. The random-effects model assumed that there was a mean population effect size for which the study-specific effect varied. Thus, we could examine interstudy heterogeneity, such as differences in the study design type and definitions of success, as well as complications. We used the inconsistency statistic (I^2^) to evaluate the extent of heterogeneity. An I^2^ value greater than 50% was considered to indicate substantial heterogeneity. A 2-sided test at the 5% level was defined as indicating statistical significance, as determined using Stata version 15 (Stata Corp, College Station, TX, USA). Publication bias was tested using a funnel plot and Egger’s test. We used a trim-and-fill method to provide the potential missing trials if publication bias was evident.

## Results

### Publication bias

**Fig. 2:**
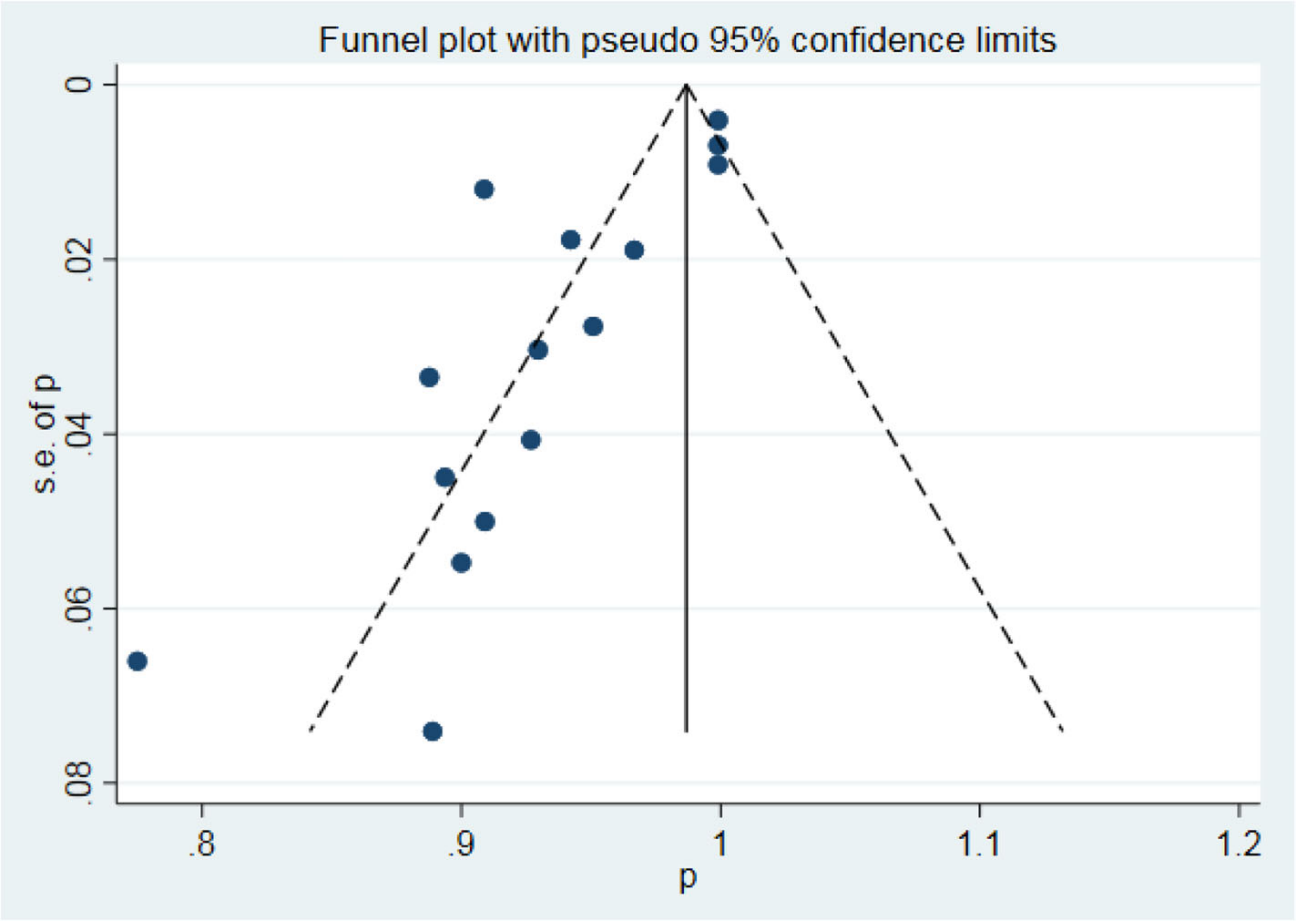
Funnel plot based on the operational success rate

**Fig. 3:**
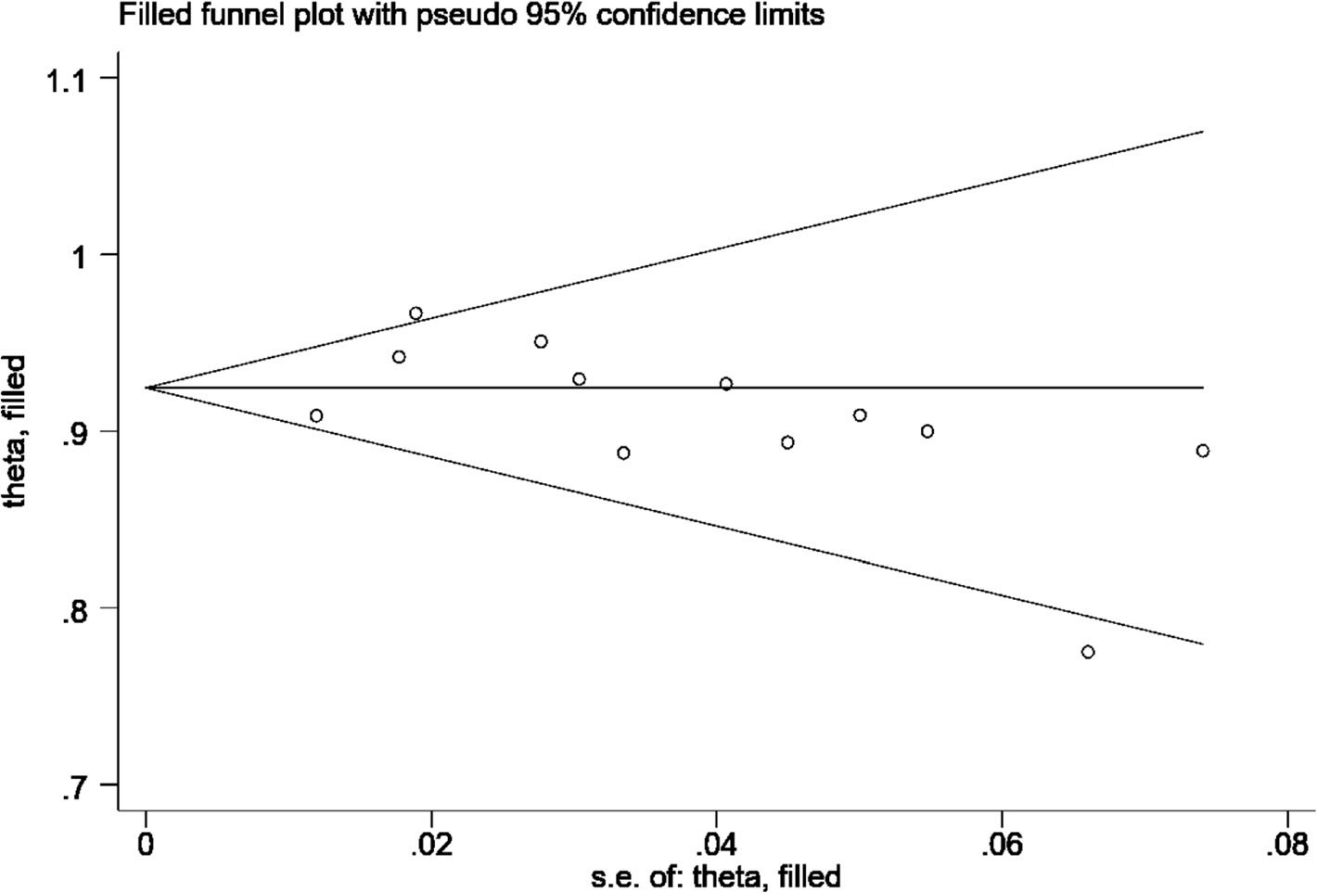
Funnel plot using the trim-and-fill method based on the operational success rate

A total of 15 studies (Table 1) investigated success and complication rates in 1368 patients and were included in the analysis. The median follow-up duration ranged from 2 months to 5 years, with the mean age of patients ranging from 2 months to 56 years. The sex rate was reported in 12 studies, including 1259 patients, 637 of whom were male. The pooled success rate was 0.95 (I^2^ = 86.2%, *P* = 0.000). Statistical evidence of publication bias was detected by a funnel plot (Fig. 2) and Egger’s and Begg’s test. The funnel plot showed funnel asymmetry, largely suggesting the presence of publication bias. P was 0.0092 in Begg’s test, and P was 0.001 in Egger’s test; both of these *P* values are less than 0.05 and suggest publication bias. We further used the trim-and-fill method to evaluate the publication bias. No trimming or filling was performed, and the 95% CI of the pooled operational success rate results was stable, which suggested an acceptable publication bias (Fig. 3).

**Table 1:** Study characteristics

N0.	First author	Year	Sample	Male	Study type	Quality score	Hospital stay (x ± s, d)	Follow-up (median year)
1	Changping Gan	2008	30	16	case series	15	3.6 ± 0.7	0.50
2	Xiang-Jun Zeng	2008	12	3	case series	15	/	/
3	Xing Quansheng	2009	21	13	case series	16	/	/
4	Kaiyu Tao	2010	61	34	case series	15	5.4 ± 1.3	1.00
5	Hua Cao	2011	18	/	case series	15	3.5 ± 1.3	0.50
6	Da Zhu	2012	40	/	case series	15	/	1.20
7	Qiang Chen	2012	89	38	case-control	7 stars	6.1 ± 0.6	1.50
8	Gui-Can Zhang	2013	71	36	case-control	8 stars	/	3.37
9	Lin Liu	2013	47	/	case series	14	4.2 ± 0.6	1.86
10	Shunmin Wang	2013	61	33	case series	15	/	2.81
11	Yu Kun Luo	2014	173	101	case-control	7 stars	9.3 ± 4.7	/
12	Yijie Hu	2014	33	15	case-control	7 stars	5.4 ± 1.5	1.67
13	Yong Sun	2016	41	16	case-control	8 stars	9.0 ± 3.0	2.30
14	WB Ou-yang	2017	581	284	case-control	8 stars	/	2.38
15	Guan-Hua Fang	2018	90	4	case-control	8 stars	4.2 ± 1.6	1.00

### Outcomes

**Fig. 4:**
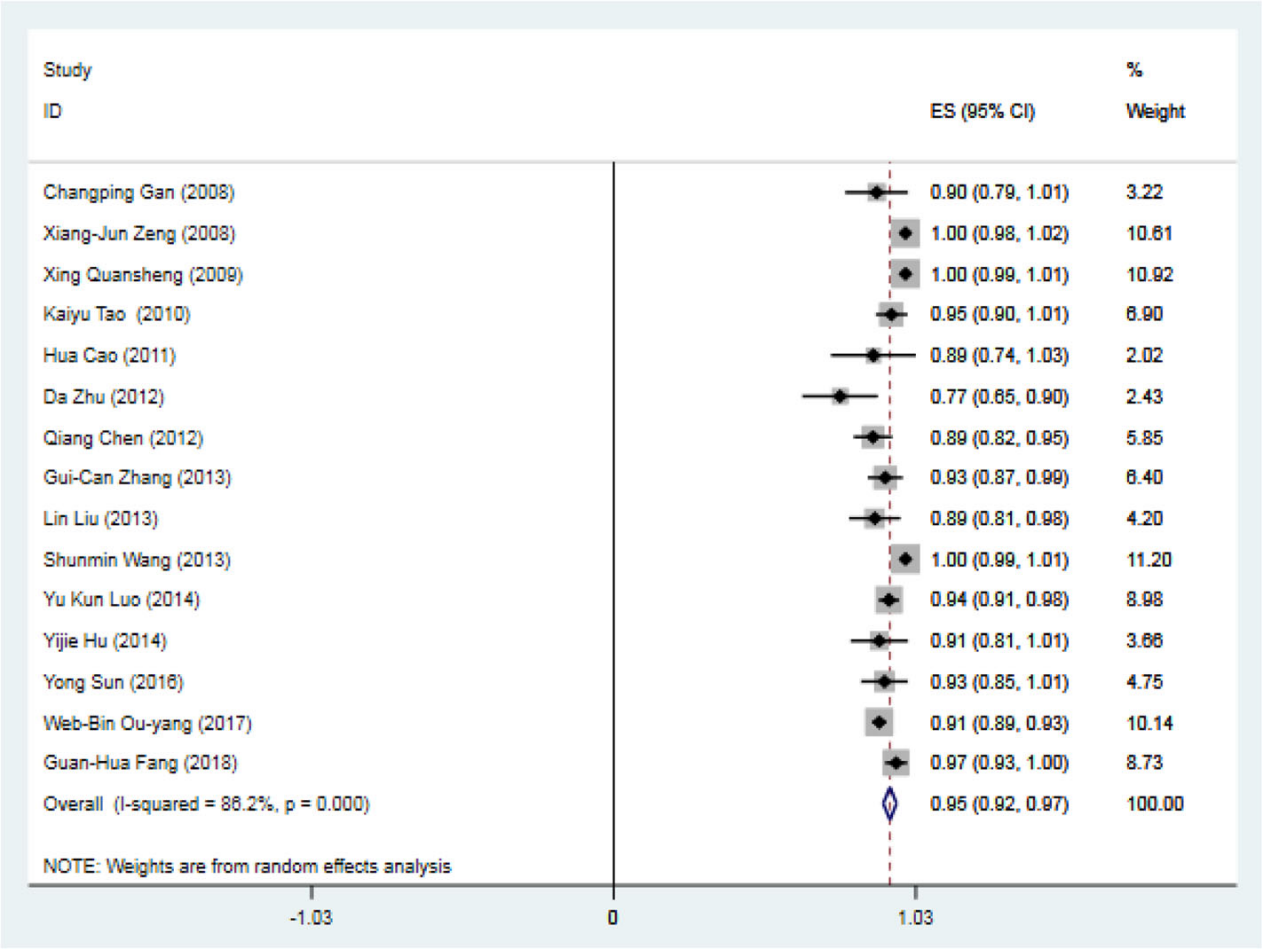
Forest plot of operational success rate

The success rate of perventricular device closure of pmVSD was high, with 11 out of 15 studies reporting a success rate of greater than 90%. Only 4 studies (sample size ranging from 12 to 61) reported a success rate less than 90%. The Q statistic showed evidence of substantial heterogeneity (I^2^ = 86.2%, *P* = 0.000), and we chose the random-effects model. The pooled estimate of the overall success rate of device closure in the 15 studies was 0.95 (95% CI: 0.92–0.97) (Fig. 4).

**Fig. 5:**
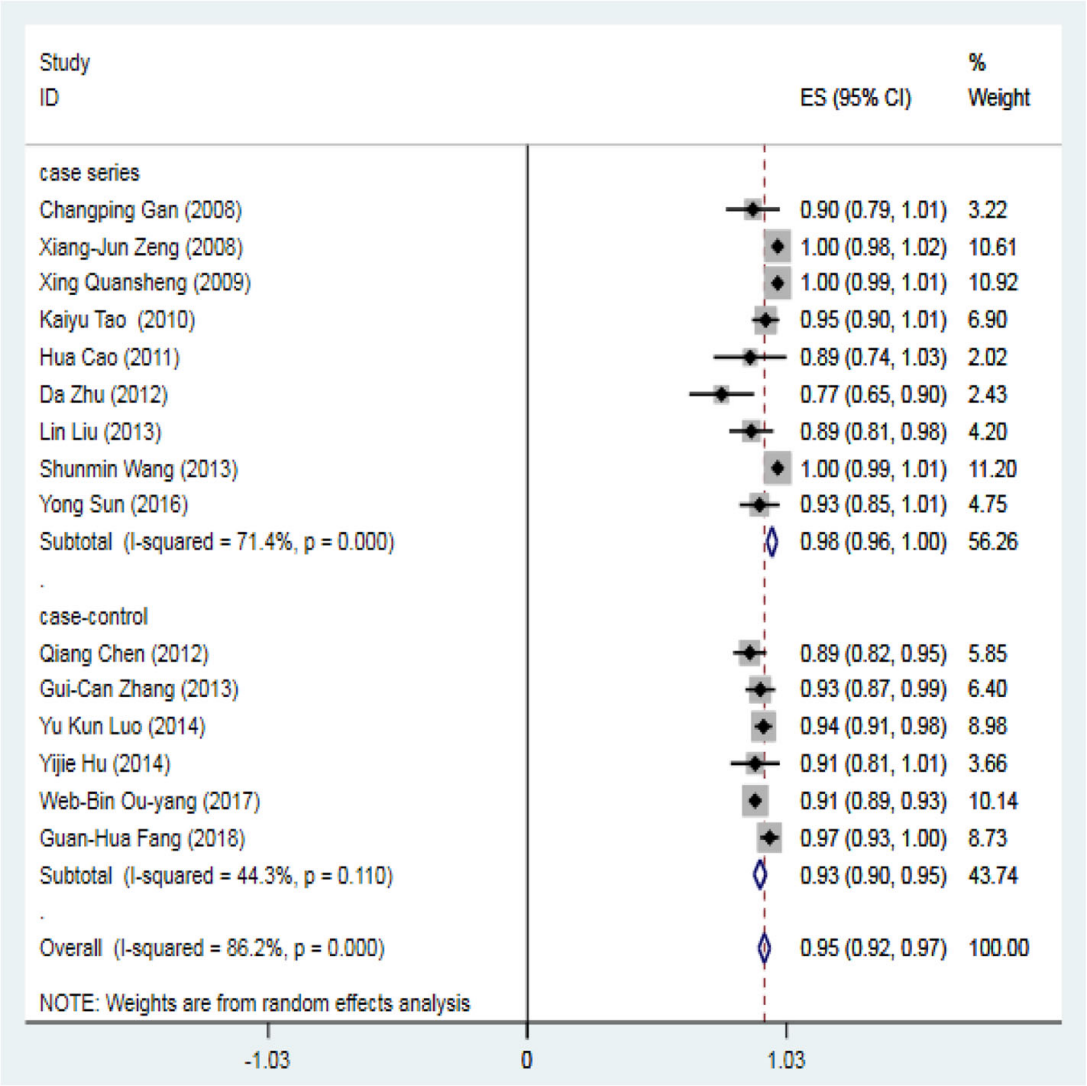
Forest plot of operational success rate stratified by study type

To explore the heterogeneity, we performed a subgroup analysis by study type and divided all studies into the case series and case-control groups (Fig. 5). The I^2^ was 44.3% in the case-control group and 71.4% in the case series group. Compared with 86.2%, both groups showed a lower I^2^, and this result suggests that the study type may be a source of heterogeneity. Meta-regression analysis indicated no significant correlation between the success rate and the following factors: publication year, sample size, study type, mean age, mean VSD size, male prevalence and TTE/TEE guidance (all *P* > 0.05). A sensitivity analysis results were further performed. Excluding 3 studies with a 100% success rate, the pooled success rate was 0.92 (95% CI: 0.90–0.94, I^2^ = 31.1%, *P* = 0.142). The heterogeneity was lower after excluding studies with a 100% success rate, and no significant difference in the success rate was found.

**Fig. 6:**
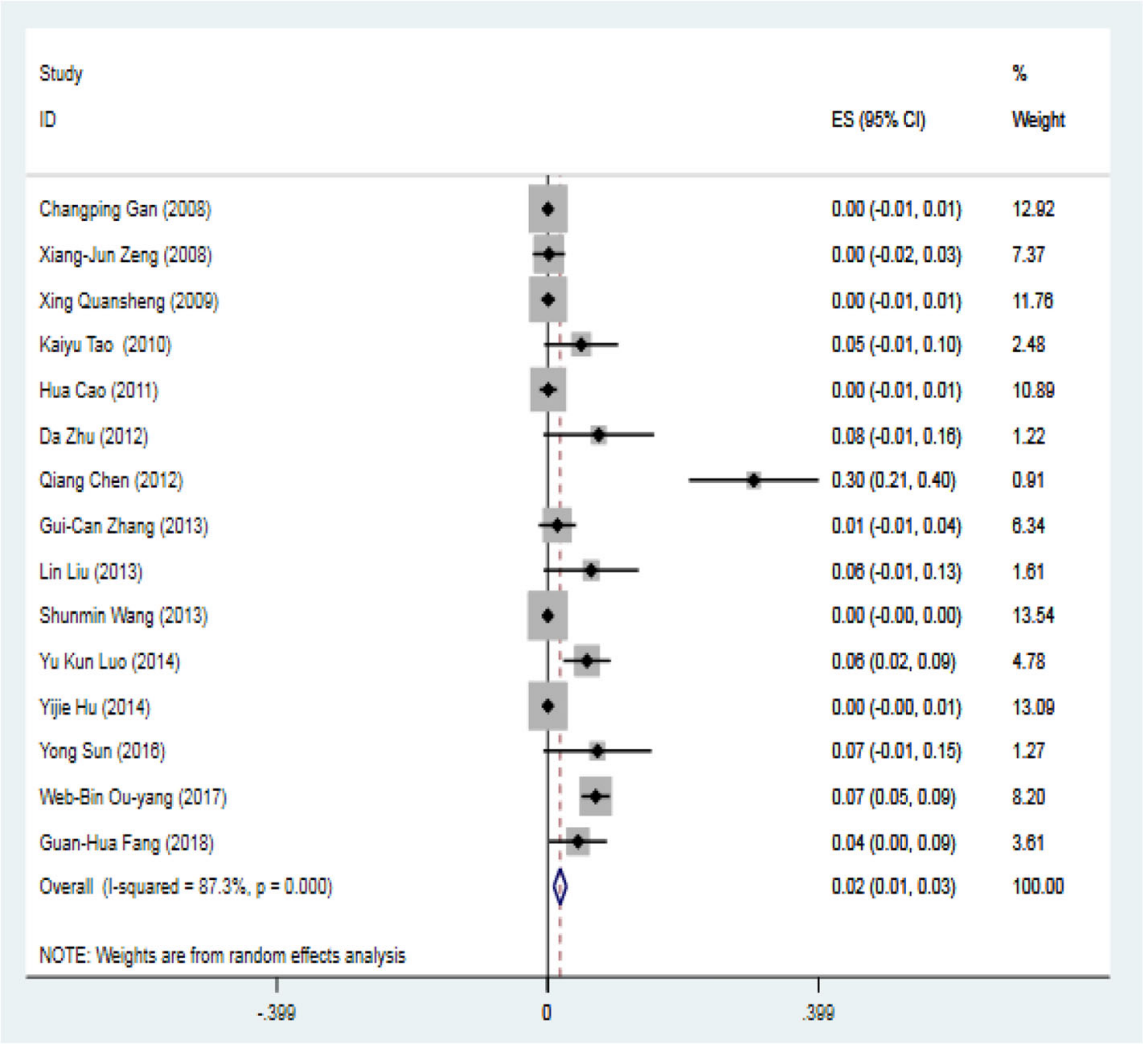
Forest plot of total postoperative residual shunting rate

The most common minor complication was residual shunting, documented in 95 subjects among the 15 studies with 1368 patients. The pooled rate of postoperative residual shunting was 0.02 (95% CI: 0.01–0.03, I^2^ = 87.3%, *P* < 0.001) (Fig. 6) The pooled rate of follow-up residual shunting was 0.001 (95% CI: − 0.001-0.002, I^2^ = 30.5%, *P* = 0.126).

A total of 80 patients were converted to conventional surgical repair. The reasons for conversion to conventional surgical repair included significant residual shunting (36.4%), mild to significant aortic regurgitation (35.2%), severe arrhythmia (11.4%), failure to establish a path (9.1%), and mild to significant tricuspid regurgitation (8.0%). Few patients required blood transfusion with a median rate of 0% (95% CI:-0.003–0.005, I^2^ = 0, *P* = 0.739), representing 4/284 patients in 8 studies. The pooled rates of intraoperative, postoperative and follow-up severe complications are shown in Table 2.

**Table 2:** The pooled rate of severe intra-operative, postoperative and follow-up complications

Pooled events	Events(n)	%	Included studies	Incidence(95%CI)	Heterogeneity(I^2^)	*P*
Total severe complications	109	100	15	0.074 (0.046–0.102)	78.00	0.00
Intra-operative	88	80.73	15	0.050 (0.028–0.071)	71.00	0.00
significant residual shunt	32	29.36	15	0.006 (0.001–0.011)	50.00	0.01
newly AR	31	28.44	15	0.008 (0.002–0.014)	48.30	0.02
AVB	10	9.17	15	0.003 (0.000–0.005)	0.00	0.94
failure in establishing track	8	7.33	15	0.001(−0.001–0.003)	0.00	0.90
newly TR	7	6.42	15	0.001(− 0.001–0.003)	0.00	0.93
Postoperative	12	11.01	15	0.000(−0.000–0.000)	0.00	0.58
newly TR	1	0.92	15	0.000(−0.000–0.000)	0.00	1.00
newly AR	0	0.00	15	0.000(−0.000–0.000)	0.00	1.00
AVB	5	4.59	15	0.000(−0.000–0.000)	0.00	0.98
occluder dislogement	2	1.83	15	0.000(−0.000–0.000)	0.00	1.00
second operation	4	3.67	15	0.000(−0.000–0.000)	0.00	1.00
Follow-up period	9	8.26	15	0.000(−0.000–0.000)	0.00	0.49
AVB	3	2.75	15	0.000(−0.000–0.000)	0.00	0.99
AR	5	4.59	15	0.000(−0.000–0.000)	0.00	0.98
TR	0	0.00	15	0.000(−0.000–0.000)	0.00	1.00
Reisidual shunt	1	0.92	15	0.000(−0.000–0.000)	26.90	0.18

All residual shunt, AR, TR listed in this table were all above mild. AVB listed in this table included Mobitz type II atrioventricular block and complete AVB

*TR* tricuspid regurgitation, *AR* aortic regurgitation, *AVB* atrioventricular block

## Discussion

Perventricular device closure is a common treatment for VSD. The first real off-pump perventricular device closure of VSD was conducted in animal experiments in 1997 under TEE guidance and then applied in an infant with muscular VSD [28]. Subsequently, perventricular device closure of pmVSD was first reported in 2004 [29]. Recently, this technology has been widely applied in China. However, perventricular device closure of pmVSD is not applied worldwide due to safety concerns, especially concerns of heart block. Through this systematic review, we have attempted to evaluate the efficacy and safety of this technology.

The included studies were 8 case series and 7 case-control studies with a high quality and acceptable publication bias. The invasive intervention limited the blinding of the participants and personnel, and this contributed to the lack of RCTs. We contributed the bias to the following factors: first, the different study designs; second, the lack of multicenter studies in this analysis and the different patient selection criteria among the single centers; and third, the increased likelihood of studies with promising results being accepted and published.

We defined operational success as patients without fatal or severe early-term or late-term complications requiring reoperation. The pooled success rate of perventricular device closure was 0.95 (95% CI: 0.92–0.97, I^2^ = 86.2%, *P* = 0.000), including 15 studies with 1368 patients. The subgroup analysis suggested that the study type may be a source of heterogeneity. Furthermore, no uniform patient inclusion criteria were applied in all medical centers. However, only patients with isolated pmVSD were included, and patients with other coexisting cardiac anomalies, severe pulmonary hypertension, or significant aortic prolapse and newborns or young infants with a large VSD were excluded. The subaortic rim was required to be greater than 1–2 mm. The VSD size ranged from 4 to 12 mm. There was no correlation between the operational success rate and the following factors: publication year, sample size, study type, mean age, mean VSD size and TTE/TEE guidance, which indicates the short learning curve and easy promotion of this technology. Compared with conventional surgical repair, there is no need for cardiopulmonary bypass (CPB) in perventricular device closure. Compared with the transcatheter approach, the perventricular approach provides direct access and facilitates manipulation of the device position and orientation during device deployment. We attributed this to the shorter delivery path. A shorter delivery path also minimizes the risk of intracardiac structural damage due to catheter friction and rubbing. Thus, for experienced cardiac surgeons, the learning curve is short, and the promising prospects of this technology are easily promoted.

The pooled rate of postoperative residual shunting was 0.02 (95% CI: 0.01–0.03, I^2^ = 87.3%, *P* = 0.00). However, most of the shunts disappeared during the follow-up period, and the pooled follow-up rate of residual shunting was 0.00 (95% CI: 0.000–0.000, I^2^ = 30.5%, *P* = 0.00). Only 1 case of mild residual shunting during the follow-up period was observed. This change means that most residual shunts disappeared naturally during the follow-up period. Endothelialization finished several weeks after the operation, covering the surface of the device and forming neointima, thereby fully closing the residual shunt [30].

The pooled rate of severe intraoperative complications was 0.050 (95% CI: 0.028–0.071, I^2^ = 71.0%, *P* = 0.000). Patients with severe intraoperative complications, including significant residual shunting, mild to significant aortic regurgitation, severe arrhythmia, failure to establish a path and mild to significant tricuspid regurgitation, were all converted to conventional surgical repair under CPB. Significant residual shunting and mild to significant aortic regurgitation were the most common reasons for conversion. The incidence rates of severe arrhythmia, failure to establishing a path and mild to significant tricuspid regurgitation were low in perventricular device closure of pmVSD. Hu and his coworkers contributed approximately 10% of transthoracic device closure (TTDC) conversion to conventional surgical repair to unsuitable occluders, as all complications were resolved by removing the occluder [22]. A lack of multiple attempts with different types and sizes of occluders may also be a reason for conversion. Thus, among selected studies, the rate of conversion to surgical repair may also be identical. Upon the occurrence of complete atrioventricular block (cAVB), significant residual shunting (> 2 mm), new aortic regurgitation, or mild to significant tricuspid regurgitation, the procedure was converted to conventional surgical repair with CPB. Most complications disappeared after removal of the occluder, suggesting the importance of choosing a suitable occluder type and size. Asymmetrical and symmetrical occluders were the most widely used occluders and were selected for TTE/TTE-measured defect-to-aortic valve rims < 2 mm and ≥ 2 mm. The occluder size was selected according to the pmVSD diameter and was larger than the pmVSD by 1–2 mm. Failure to establish a path was reported in 5 studies, with a pooled rate of 0.000 (95% CI: − 0.000-0.000, I^2^ = 0.0%, *P* = 0.901). The precondition of establishing a path is finding a suitable puncture site perpendicular to the plane of the VSD. Surgeons mostly determine the puncture site by depressing the right ventricular free wall with an index finger to find the strongest pulsatory site under continuous TEE/TTE guidance. Unsuitable puncture results in the failure to establish a path.

The pooled rate of severe postoperative complications was 0.000 (95% CI: 0.000–0.000, I^2^ = 71.0%, *P* = 0.000). A total of 4 patients required a second operation, including 1 for occluder dislodgement and 3 for cAVB. Another patient with cAVB recovered a sinus rhythm and did not undergo a second operation or permanent pacemaker. Occluder dislodgement may be a procedure-related complication caused by a lack of experience with TTDC. In other cases of postoperative arrhythmia mentioned in the enrolled studies, a sinus rhythm was recovered within 48–72 h after surgery. This finding may be attributable to early procedure-related inflammation or the limited number of cases [18]. Only one patient experienced new mild tricuspid regurgitation, which disappeared during the follow-up period. No cases of new mild or significant aortic regurgitation were observed. The pooled rates of aortic regurgitation and tricuspid regurgitation were both 0.000 (95% CI: 0.000–0.000, I^2^ = 0.0%, *P* = 1.0). This promising result may be attributable to suitable occluder selection or the limited number of cases in this meta-analysis.

The pooled rate of severe complications in the follow-up period was 0.000 (95% CI: − 0.000-0.000, I^2^ = 0.0%, *P* = 0.487), including 3 cases of late cAVB, 5 cases of mild aortic regurgitation, and 1 case of residual shunting (> 2 mm). The above 3 patients with late cAVB recovered a sinus rhythm spontaneously or after steroid therapy. However, previous reports have emphasized that once late-onset cAVB occurs, a permanent pacemaker is the only cure for cAVB, which is in contrast to the above findings [31, 32]. One possible explanation may be that the conduction system was recently affected by the device-related inflammatory response or scar formation and the patients came to hospital for therapy immediately.

The mechanism of cAVB remains unclear. It is possible that occluder devices may cause an initial inflammatory response with subsequent formation and fibrosis in the conduction system [14]. Progressive device flattening may also be a mechanism for the development of cAVB, according to Butera G’s hypothesis [33]. Compared with the transcatheter approach, perventricular device closure involves a shorter path and thus avoids friction and rubbing of the conduction system and the subsequent inflammation. Meta-regression analysis indicated no significant correlation between early/late cAVB and the following factors: publication year, sample size, study type, mean age, mean VSD size, male prevalence, occluder-VSD size difference and TTE/TEE guidance (all *P* > 0.05). It is still a challenge to completely avoid cAVB given the surrounding anatomical structures in pmVSD; thus, precautions with suitable device selection (both type and size) are paramount. Certain devices have already been approved in some countries for use in pmVSD closure. No one type of occluder is suitable in all cases of VSD; thus, progressive improvements of these devices are also necessary.

Aortic regurgitation is another severe complication of perventricular device closure due to the short subaortic rim of pmVSD and the use of unsuitable occluders. Only 5 cases of mild aortic regurgitation were observed during the follow-up period. The pooled rate of aortic regurgitation in the follow-up period was 0.000 (95% CI: − 0.000-0.000, I^2^ = 0.0%, *P* = 0.982). This result show that the incidence of aortic regurgitation in the follow-up period was low, emphasizing the importance of accurately evaluating the subaortic rim and choosing a suitable occluder.

## Conclusion

Perventricular device closure may be an alternative to conventional surgical repair in selected patients with pmVSD. This meta-analysis proves perventricular device closure of pmVSD to be safe and effective. The success rate was stable regarding the publication year and sample size and suggested the short learning curve of this technology and its prospects for wide application. The incidence of severe arrhythmia, especially cAVB, was low. These good results may be limited by the number of enrolled patients, and more detailed observations in a larger sample are required for further analysis.

### Study limitations

First, heterogeneity existed in this meta-analysis, largely due to differences in study design. Second, several studies enrolled in the meta-analysis did not provide sufficient information regarding major outcomes. Some studies reported all cases of arrhythmia, whether severe or minor, while others only reported cases of severe arrhythmia. Difficulties were encountered when classifying complications into transient and permanent subgroups, as most studies included patients who did not receive or report all of the appropriate follow-up end-points. The follow-up period was different in each study. It is difficult to define transient or permanent; we only enrolled cases reported at the final follow-up review as being permanent and recorded all other cases as being transient. Most studies did not provide subarterial rim data or the type of occluder used in patients. Third, this analysis included case series and case-control studies but no randomized controlled studies. Finally, there was acceptable publication bias in this study.

## Data Availability

Data sharing not applicable to this article as no data sets were generated or analyzed during the current study.

## Abbreviations

AR: Aortic regurgitation
AVB: Atrioventricular block
cAVB: Complete atrioventricular block
CPB: Cardiopulmonary bypass
NOS: Newcastle-Ottawa Scale
pmVSD: Perimembranous ventricular septal defects
TEE: Transesophageal echocardiography
TR: Tricuspid regurgitation
TTDC: Transthoracic device closure
TTE: Transthoracic echocardiography
